# Canine Mammary Tumor Cell Lines Derived from Metastatic Foci Show Increased RAD51 Expression but Diminished Radioresistance via p21 Inhibition

**DOI:** 10.3390/vetsci9120703

**Published:** 2022-12-17

**Authors:** Kei Shimakawa, Kazuhiko Ochiai, Sachi Hirose, Eri Tanabe, Masaki Michishita, Motoharu Sakaue, Yasunaga Yoshikawa, Masami Morimatsu, Tsuyoshi Tajima, Masami Watanabe, Yoshikazu Tanaka

**Affiliations:** 1 Laboratory of Veterinary Hygiene, School of Veterinary Science, Nippon Veterinary and Life Science University, Tokyo 180-8602, Japan; 2 Research Center for Animal Life Science, School of Veterinary Science, Nippon Veterinary and Life Science University, Tokyo 180-8602, Japan; 3 Laboratory of Veterinary Pathology, School of Veterinary Science, Nippon Veterinary and Life Science University, Tokyo 180-8602, Japan; 4 Laboratory of Anatomy II, Department of Veterinary Medicine, Azabu University, Sagamihara 252-5201, Japan; 5 Laboratory of Veterinary Biochemistry, School of Veterinary Medicine, Kitasato University, Aomori 034-8628, Japan; 6 Laboratory of Laboratory Animal Science and Medicine, Graduate School of Veterinary Medicine, Hokkaido University, Sapporo 060-0818, Japan; 7 Department of Veterinary Pharmacology, School of Veterinary Science, Nippon Veterinary and Life Science University, Tokyo 180-8602, Japan; 8 Department of Urology, Graduate School of Medicine, Dentistry and Pharmaceutical Sciences, Okayama University, Okayama 700-8558, Japan

**Keywords:** canine, DNA damage repair, mammary tumor, p21, RAD51

## Abstract

**Simple Summary:**

Canines have a high incidence of mammary tumors, which is a major problem in the veterinary field. Approximately half of canine mammary tumors are malignant and metastasize to nearby lymph nodes. In this study, we analyzed the expression levels of RAD51, a DNA homologous recombination repair-related molecule, in canine mammary tumor cell lines derived from the primary tumor CHMp and metastatic tumor CHMm, which were established from the same individual. The RAD51 expression level in CHMm was higher than that in CHMp. However, CHMp was more resistant than CHMm to irradiation and formed functional Rad51 foci, which form at DNA lesions for homologous recombination repair. The cell cycle regulator p21 was expressed upon radiation exposure in CHMp cells, but not in CHMm cells, indicating that CHMm did not induce cell cycle arrest after irradiation. These results suggest that the radioresistance of CHMm, with high RAD51 expression, is lower than that of CHMp.

**Abstract:**

Due to the high incidence of mammary tumors in dogs, it is important to elucidate the pathogenesis of these tumors in veterinary medicine. Radiation therapy is often used to treat mammary tumors that target DNA lesions. RAD51 is a key molecule that repairs DNA damage via homologous recombination. We examined the relationship between RAD51 expression and radiosensitivity in mammary tumor cell lines. CHMp and CHMm from the same individual were selected based on the differences in RAD51 expression. The radiosensitivity of both cell lines was examined using MTT and scratch assays; CHMm, which has high RAD51 expression, showed higher sensitivity to radiation than CHMp. However, the nuclear focus of RAD51 during DNA repair was formed normally in CHMp, but not in most of CHMm. Since irradiation resulted in the suppression of cell cycle progression in CHMp, the expression of p21, a cell cycle regulatory factor, was detected in CHMp after 15 Gy irradiation but not in CHMm. These results indicate that functional expression is more important than the quantitative expression of RAD51 in canine mammary tumor cells in response to DNA damage.

## 1. Introduction

RAD51 is a highly structurally conserved molecule in organisms ranging from *Escherichia coli* to higher mammals and plays an important role in maintaining genome stability [1,2]. RAD51 forms a complex with breast cancer susceptibility gene 2 (BRCA2) upon DNA damage, such as DNA double-strand breaks (DSB) for homologous recombination (HR) repair [3,4]. HR is catalyzed by RAD51 recombinase, which forms a nucleoprotein filament on resected single-stranded DNA (ssDNA) at the damage site, and mediates the pairing of homologous DNA sequences and strand invasion [5]. Disruption of the interaction between RAD51 and BRCA2 leads to genomic instability and the development of mammary tumors [6,7,8]. RAD51 and BRCA2 are essential for survival, RAD51 knockout mouse embryos are lethal, and dysfunctional RAD51 also results in abnormal spermatogenesis [9,10].

Canines have a high incidence of mammary tumors [11], and several mutations have been detected in BRCA2 and RAD51 [12,13,14,15,16]. In canine studies, RAD51 forms nuclear foci during DNA damage and may function in damage repair [17]. Therefore, the maintenance of genomic stability by RAD51 and BRCA2 plays a potential role in the prevention of tumorigenesis in dogs. Although RAD51 and BRCA2 are essential for maintaining genomic stability, their increased expression has been reported in lymph node metastases of canine mammary adenocarcinomas [18,19]. Some reports have suggested that overexpression of RAD51 induces radioresistance in human cancer tissues [20,21] and enhances metastasis in human breast cancer cell lines [22]. These phenomena indicate that maintenance of genomic stability is dependent upon tight regulation of RAD51 and BRCA2, key molecules of HR. In 2006, Uyama et al. established eight cell lines from primary and metastatic foci in four canines with mammary tumors [23], respectively, which have been used in subsequent studies [24,25,26]. Several reports on the radiosensitivity and radioresistance of canine cancer cell lines exist. Squamous cell carcinoma, fibrosarcoma, and hemangiocytoma cell lines showed radiation sensitivity, with a survival rate of less than 10% after 5.76 ± 0.33 Gy of γ-radiation [27]. Cancer stem cells derived from a canine lung adenocarcinoma cell line survived for 7 days of over 10 Gy of radiation dose [28]. However, the relationship between radiosensitivity and radioresistance in canine mammary tumor cell lines, focusing on DNA HR repair-related molecules, has not yet been analyzed.

In this study, we investigated the role of HR-related molecules in canine mammary tumors. We selected two cell lines with low and high RAD51 expression from eight mammary tumor-derived cell lines. These cell lines were examined for radiosensitivity and the relationship between RAD51 expression and function. Additionally, we analyzed the relationship between the expression levels of the cell cycle control molecules p21 and RAD51.

## 2. Materials and Methods

### 2.1. Cells and Cell Culture

HeLa cells were purchased from the American Type Culture Collection (ATCC, Manassas, VA, USA). Canine mammary gland tumor (CMT) cell lines were established and characterized in previous studies. CHMp cells were collected from the primary nest of clinical stage IV mammary adenocarcinoma, and CHMm cells were collected from pleural effusions. These cell lines were established from the same individual 12-year-old mixed female dog [23]. HeLa cells were maintained in Dulbecco’s modified Eagle’s medium (FUJIFILM Wako Pure Chemical Corporation, Osaka, Japan), and CMT cell lines were maintained in RPMI1640 medium (FUJIFILM Wako Pure Chemical Corporation), supplemented with 10% fetal bovine serum (FBA), penicillin, and streptomycin (FUJIFILM Wako Pure Chemical Corporation), and incubated at 37 °C in a 5% CO_2_ atmosphere.

### 2.2. Double Thymidine Block

To synchronize the G_0_ phase, sub-confluent CHMp and CHMm cells were treated with 2 mM thymidine for 18 h. After washing twice in phosphate-buffered saline (PBS), the cells were cultured in fresh DMEM with 10% FBS for 9 h and then treated with 2 mM thymidine for another 18 h. After washing twice in PBS, the cells were incubated in DMEM containing 10% FBS and collected at the designated time points for assays.

### 2.3. Western Blot Analysis

Cells were lysed with mammalian lysis buffer (Promega Corporation, Madison, WI, USA) supplemented with a protease inhibitor cocktail (Promega Corporation). Protein concentrations were determined using a BCA protein assay kit (Nacalai Tesque, Kyoto, Japan). The protein extract from the cells (10 μg) was mixed with 2× loading buffer and separated on 5–20% gradient SDS-PAGE (Dream Realization and Communication: DRC). The separated proteins were transferred onto polyvinylidene difluoride (PVDF) membranes. Membranes were blocked with EzBlock Chemi reagent (ATTO Corporation, Amherst, NY, USA) for 1 h at 25 °C. Western blot analysis was performed using the following primary antibodies for 16 h at 4 °C: rabbit polyclonal anti-RAD51 (1:1000; cat. no. sc-8349, Santa Cruz Technology, Dallas, TX, USA), rabbit polyclonal anti-Halo (1:1000; cat. no. G9281, Promega Corporation), rabbit polyclonal anti-p21 (1:1000; cat. no. 10355-1-AP, Proteintech, Rosemont, IL, USA), and mouse monoclonal anti-α-tubulin (1:2000; cat. no. 013-25033, FUJIFILM Wako Pure Chemical Corporation). Horseradish peroxidase-conjugated secondary antibodies (1 h at 25 °C) and EzWestLumi plus (ATTO Corporation) were used to detect the antibody-bound proteins.

### 2.4. Induction of DNA Damage

Irradiation of CHMp and CHMm cell lines was carried out using a Faxitron CP160 irradiator (Faxitron X-ray Corporation, Tucson, AZ, USA) at 5 Gy/min, for a total maximum dose of 15 Gy. Doxorubicin stimulation was applied to the CHMp and CHMm cell lines at suitable concentrations of doxorubicin (FUJIFILM Wako Pure Chemical Corporation) in the medium.

### 2.5. MTT Assay

The effects of irradiation on CHMp and CHMm cells were evaluated using MTT assay. Cells were plated in 96-well plates at a density of 1.5 × 10^3^ cells/well. After 24 h, the cells were irradiated at various intensities (0, 1, 2.5, 5, 10, and 15 Gy) and incubated for 72 h at 37 °C. At the end of the treatment period, 7.5 μL MTT (Dojindo, Kumamoto, Japan, 5 mg/mL PBS) was added to each well. The cells were incubated for 4 h at 37 °C. Colored crystals of the produced formazan were dissolved in 150 μL DMSO. The purple-blue formazan formed was measured at 560 nm using a MULTISKAN FC (Thermo Fisher Scientific, Inc. Waltham, MA, USA). The optical density of each sample was compared with the optical density of the control, and graphs were plotted using the SkanItTM Software ver. 4.1 (Thermo Fisher Scientific, Inc.). IC_50_ values were obtained from the inhibition curve plots.

### 2.6. Tumor Cell Migration Assay

Inhibition of CHMp and CHMm cell migration after irradiation was assessed using a wound healing assay [29]. CHMp and CHMm cells were seeded in RPMI-1640 medium, supplemented with 10% FBS, penicillin, and streptomycin, and grown in confluent monolayers in 6-well plates at a density of 4 × 10^5^ cells/well for 24 h. A single scratch wound was created using a sterile micropipette tip. Subsequently, cell debris was removed by washing the plates twice with PBS, and the cell culture medium was refreshed with 1% FBS. The cells were cultivated for up to 24 h. Three independent experiments were performed and the area of cell migration was examined using an inverted microscope (DM IL LED, Leica Microsystems, Wetzlar, Germany) and quantified using ImageJ ver. 1.53e software (National Institutes of Health, Bethesda, MD, USA).

### 2.7. Immunostaining of RAD51 Foci and Microscopy

CHMp and CHMm cell monolayers (80% confluent) were cultured on coverslips (Matsunami Glass, Bellingham, WA, USA). After 24 h, the CHMp and CHMm cell lines were irradiated with 15 Gy. The cells were immediately returned to the tissue culture incubator and fixed with 4% paraformaldehyde for 6 h after the irradiation. After permeabilization with 0.3% Triton X-100 in PBS, cells were incubated with H-92, a polyclonal antibody against human RAD51 (1:250; cat. no.sc-8349, Santa Cruz Technology), followed by Alexa Fluor-568-conjugated goat anti-rabbit IgG (Molecular Probes, Eugene, OR, USA) [17]. RAD51 foci were examined under a fluorescence microscope (Nikon Corporation, Tokyo, Japan). Images containing at least 100 cells were captured by a computer, and the number of cells containing at least 10 foci or deep red-dyed nuclei was recorded and plotted as a percentage of the total number of cells.

### 2.8. cDNA Cloning, Sequencing, and Structure Analysis of Canine p21

Canine p21 was amplified using polymerase chain reaction (PCR) with the following oligonucleotide primers: canine p21-F (5′- CGAGCTGCTGCGGGAGACGGTG-3′) and canine p21-R (5′- GTGGCAAGCAGGGTATGTACATG-3′). These primers were designed based on predicted canine p21 sequences (GenBank accession no. XM_532125.6). Total RNA was obtained from the canine kidney (Zymo Research Corp., Irvine, CA, USA). Total RNA (4 μg) was denatured at 70 °C for 10 min, cooled immediately, and reverse-transcribed using 200 units of SuperScript III (Thermo Fisher Scientific Inc.), 25 pmol of random primer, and 10 nmol dNTPs in a total volume of 20 μL at 37 °C for 50 min. PCR amplification was performed using PrimeSTAR (Takara Bio, Shiga, Japan). The PCR was conducted for 30 cycles, each consisting of denaturation at 96 °C for 30 s, annealing at 55 °C, and extension at 72 °C for 1 min. Sequence data were determined for at least five independent clones using the ABI 3730 platform (Applied Biosystems; Thermo Fisher Scientific Inc.). For the sequence analysis, human p21 (GenBank accession No. NP_000380.1) and canine p21 (GenBank accession No. BCL66287.1) were compared using the Genetyx software ver. 16 (Genetyx Corporation, Tokyo, Japan) and the InterPro classification of protein families program (https://www.ebi.ac.uk/interpro/) (accessed on 30 July 2022) [30]. dATP was added to the PCR products using a 10× A-attachment kit (Toyobo Life Science, Osaka, Japan) and cloned into the pGEM-T Easy vector (Promega Corporation). These clones were subcloned into the Halo-tagged expression vector pFN21A (Promega Corporation) containing *Sgf*I/*Pme*I sites.

### 2.9. Cell Cycle Analysis

The cells were treated with 15 Gy irradiation. Following 72 h of incubation at 37 °C, the cells were detached by trypsinization, washed twice in PBS, and fixed with 70% EtOH for 24 h at −25 °C. For analysis, 5 × 10^5^ cells were washed with PBS, resuspended in 200 μL of Muse^®^ Cell Cycle Reagent (Luminex Corp., Austin, TX, USA), and incubated for 30 min at 22 °C in the dark. Finally, the cell suspension was transferred into a new 1.5 mL tube without a cap, and the samples were analyzed using flow cytometry on a Guava^®^ Muse^®^ Cell Analyzer (Luminex Corp.).

### 2.10. Microsatellite Genotyping of CHMp and CHMm Using PCR and Amplified Fragment Length Polymorphism Analysis

Thirteen microsatellite loci were compared between CHMp and CHMm cell lines using PCR and amplified fragment length polymorphism analysis in a genetic diagnosis laboratory (http://www.kahotechno.co.jp/clinic/index.html) (accessed on 30 July 2022) [26].

### 2.11. p53 Tetramerization Reporter Assay

The p53 response element in p21WAF (GenBank accession no. U24170, nucleotides 2303–2321) and human cytomegalovirus in the pTet-splice vector (nucleotides 318–446; Clontech Laboratories Inc., Mountain View, CA, USA) [31] were inserted between the *Xho*I and *Hind*III sites in pNL1.1[Nluc] (p53RE-pNL1.1) (Promega). To measure endogenous p21 transcriptional activities, CHMp and CHMm cells were transfected with the p53RE-pNL1.1. The cells were harvested 48 h after transfection, and luciferase activity was measured using the Nano-Glo Dual-Luciferase Reporter Assay System (Promega). Luciferase activity was normalized to the value of luc2 activity from the co-transfected pGL4.51[luc2/ CMV/Neo] (Promega).

### 2.12. Statistical Analysis

Data are expressed as mean ± standard deviation. Analysis of variance with Tukey’s post hoc test was used when multiple comparisons were required. A value of *p* < 0.01 indicated a statistically significant difference.

## 3. Results

### 3.1. RAD51 Expression Was Different in Each Canine Mammary Tumor Cell Line

**Figure 1:**
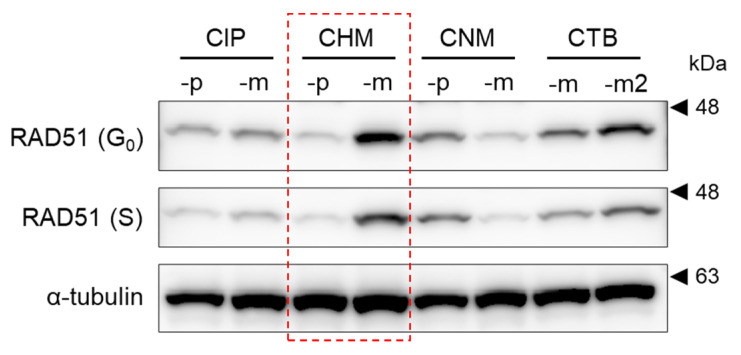
Analysis of RAD51 expression in eight canine mammary tumor cell lines. RAD51 expression status in the G_0_ and S phases in double thymidine-blocked mammary tumor cell lines was analyzed using western blotting. α-tubulin was used as a loading control. CHMp and CHMm derived from the same individual with markedly different RAD51 expressions are indicated by red dotted boxes. (The original gel figures can be found in the Supplementary Materials.).

To investigate RAD51 expression, proteins were extracted from double thymidine-blocked canine mammary tumor cell lines. A comparison of RAD51 expression levels using western blotting showed that RAD51 expression levels were different in each of the eight cell lines. Particularly, CHMp (derived from primary foci) and CHMm (derived from metastatic foci), which were established from the same individual, showed remarkable differences in both G_0_ and S phases (Figure 1).

### 3.2. Cell Survival after Irradiation

**Figure 2:**
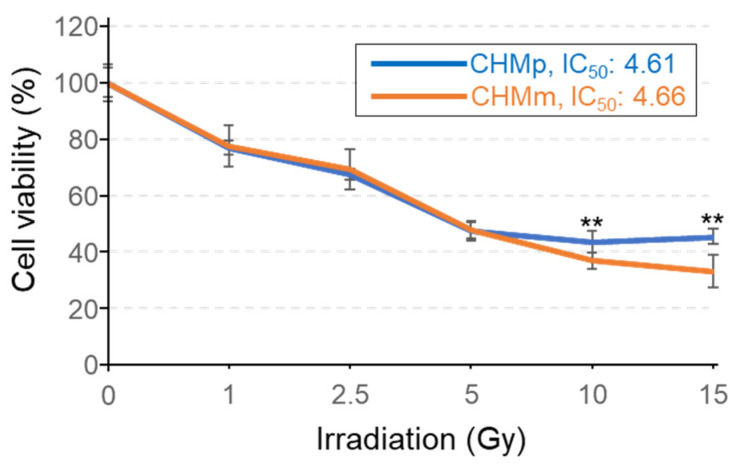
Cell survival after irradiation of CHMp and CHMm. Cells were treated with increasing intensities of radiation (0, 1, 2.5, 5, 10, 15 Gy). After 72 h, the IC_50_ values and cell viability using an MTT assay were determined by measuring the absorbance at 560 nm on a microplate reader. The values of no treatment were 100%, and the values are shown from eight independent experiments as the mean ± SD. Asterisks on top of the brackets indicate significant differences calculated using ANOVA with Tukey’s multiple-comparison test (** *p* < 0.01).

CHMp and CHMm were irradiated, and after 72 h, cell viability was evaluated using the MTT assay. IC_50_ of radiation exposure of CHMp was 4.61 Gy and that of CHMm was 4.66 Gy, which were similar; however, high dose irradiation of 10 or 15 Gy resulted in lower survival rates for CHMm (Figure 2).

### 3.3. Comparison of Migration Abilities after Radiation

**Figure 3:**
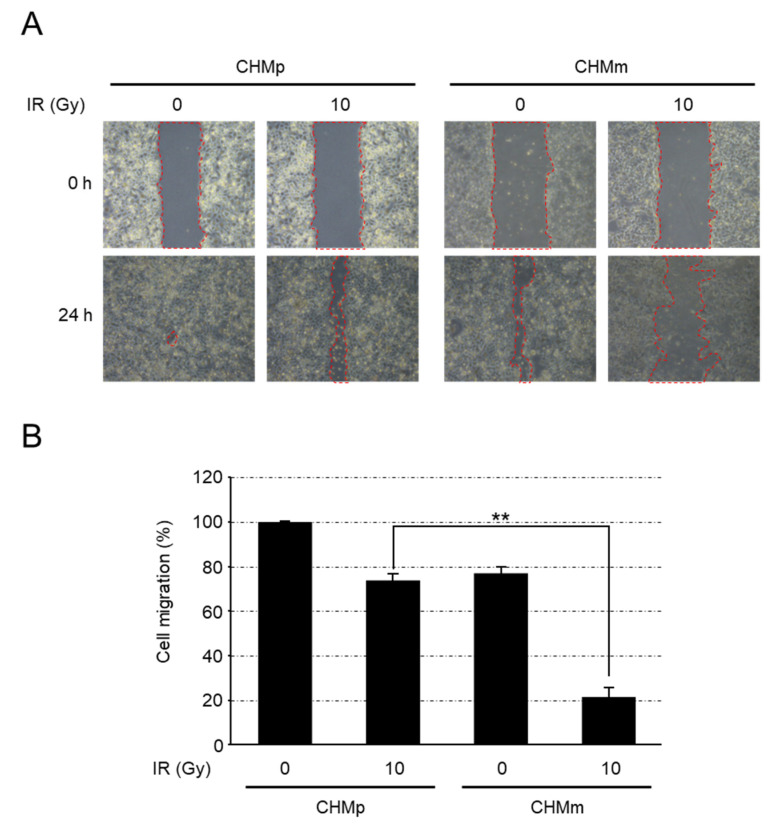
Cell migration after irradiation of CHMp and CHMm. (**A**) Migration of CHMp and CHMm cell lines that were incubated for 72 h after irradiation was assessed using a wound healing assay 24 h after scratch. Representative images of the scratched areas are shown. Microscope magnification, ×200. (**B**) Cell migration was quantified using ImageJ software. The values of cell migration of CHMp without irradiation are 100%, and the values are shown from three independent experiments as the mean ± SD. Asterisks on top of the brackets indicate significant differences calculated using ANOVA with Tukey’s multiple-comparison test (** *p* < 0.01).

Twenty-four hours after scratching without irradiation, CHMp cells recovered almost completely, whereas recovery of CHMm cells was incomplete (Figure 3A). With 10 Gy radiation treatment, the CHMp cells largely recovered from the wound after 24 h; the CHMm cells exhibited a significantly delayed wound healing ability (Figure 3B).

### 3.4. CHMm Cells Had Reduced Ability to Form RAD51 Foci after Irradiation

**Figure 4:**
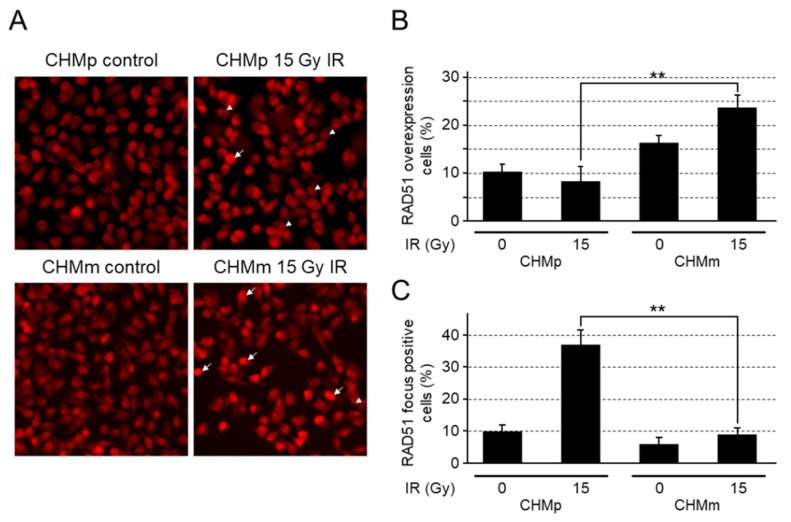
RAD51 focus formation in CHMp and CHMm. (**A**) RAD51 immunostained cells, before and after being subjected to ionizing radiation. The indicated cells were irradiated (15 Gy) and fixed 6 h after exposure to the ionizing radiation. In the panel for RAD51 foci, the nuclei of focus-forming cells are indicated by arrowheads, and deep-red-dyed nuclei are indicated by arrows. (**B**,**C**) Images containing at least 100 cells were captured by a computer, and the number of cells containing deep-red-dyed areas determined to be RAD51-overexpressing nuclei (**B**) or at least 10 foci (**C**) was recorded and plotted as a percentage of the total number of cells. The plots were generated from three independent experiments. The results are given as the mean ± SD. Asterisks on top of the brackets indicate significant differences calculated using ANOVA with Tukey’s multiple-comparison test (** *p* < 0.01).

To confirm the RAD51 function in the repair of irradiation-induced DNA damage, RAD51 focus formation assays were performed in CHMp and CHMm. CHMm cells showed red-dyed nuclei, reflecting high RAD51 expression in the absence of irradiation compared with CHMp cells; these cell numbers were further increased by irradiation (Figure 4A,B). In contrast, CHMp formed distinct RAD51 foci in the nucleus after irradiation (Figure 4A,C).

#### 3.4.1. Irradiation Does Not Lead to Cell Cycle Arrest in CHMm

**Figure 5:**
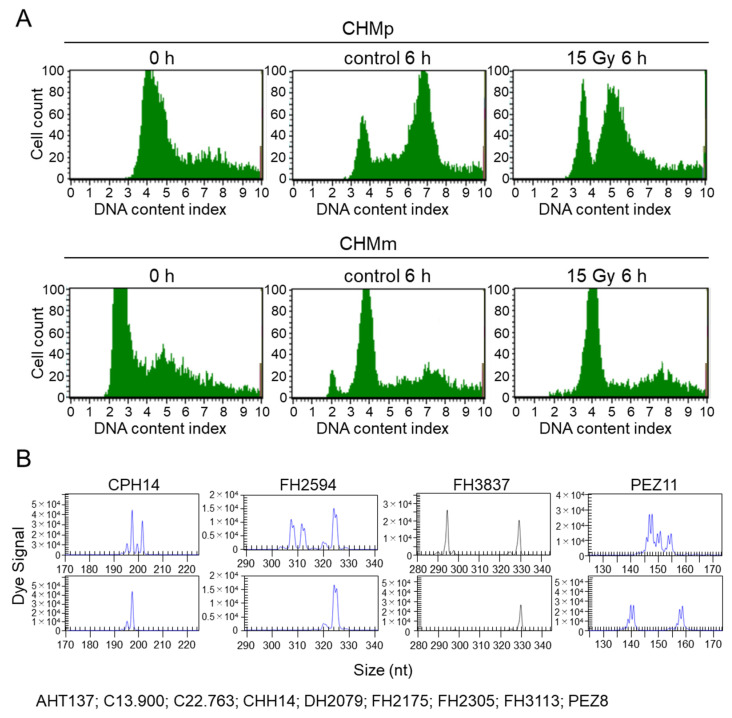
Cell cycle profile after irradiation and microsatellite profile of CHMp and CHMm. (**A**) FACS analysis using PI staining was performed after 15 Gy irradiation. (**B**) Microsatellite analysis of cell lines established from the same origins. Thirteen microsatellite loci were compared between CHMp (upper panel) and CHMm (lower panel) cells. Four loci (CPH14, FH2594, FH3837, and PEZ11) were different, but the other nine loci shown under the panels were identical between these two cell lines.

To verify the effect of irradiation-induced cell cycle arrest on CHMp and CHMm cells, the cell cycle was synchronized using a double thymidine block, followed by irradiation with 15 Gy, and the cell cycle was observed 6 h later. The DNA content of CHMm, which was synchronized to the G0 phase, was lower than that of CHMp (Figure 5). Inhibition of the increase in intracellular DNA content was observed in the irradiated group compared to that in the non-irradiated group for CHMp, but not for CHMm (Figure 5A).

#### 3.4.2. Analysis of Cell Identity Using Microsatellite Analysis

Thirteen microsatellite loci were compared between CHMp and CHMm strains. Four microsatellite loci, PEZ11, FH3837, CPH14, and FH2594, were different between the CHMp and CHMm cell lines, while the other nine were identical (Figure 5B).

#### 3.4.3. Cloning and Structural Analysis of Canine PALB2

**Figure 6:**
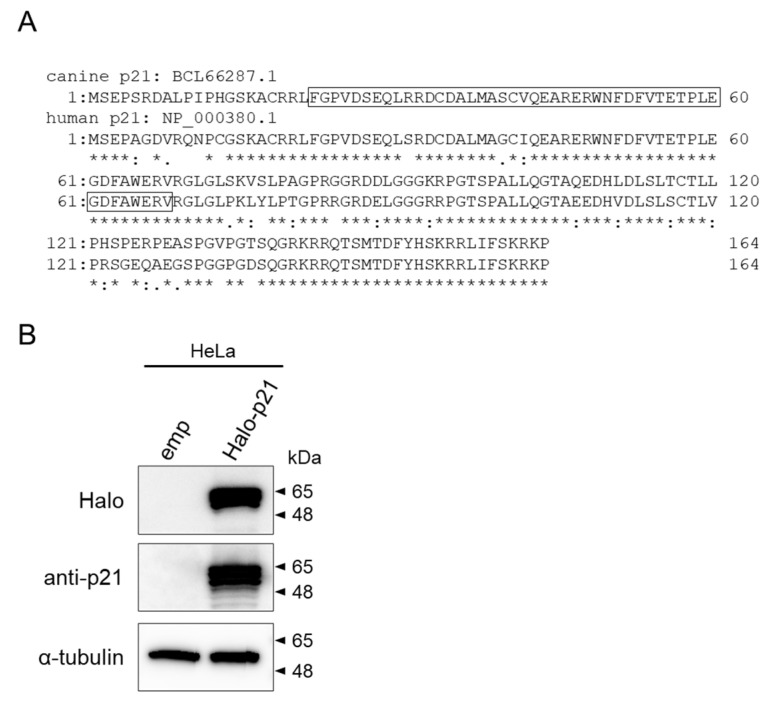
Structure analysis of canine p21 and cross-reactivity of anti-p21 antibody. (**A**) The amino acid sequence of canine p21 (BCL66287.1) was aligned with that of the human p21 (NP_000380.1) using ClustalW. Residues are denoted as full conservation (*), conservation of the strong amino group (:), or conservation of the weak amino group (·). The conserved functional domain of cyclin-dependent kinase inhibitor predicted using InterPro is boxed. (**B**) Western blot analysis using anti-Halo or human p21 antibodies. Cell lysates from Halo-tagged p21 forced expressed HeLa cells were electrophoresed using 5–20% gels. α-tubulin was used as a reference protein for the normalization of protein loading. (The original gel figures can be found in the Supplementary Materials.).

The ORF of canine p21 cDNA determined in this study (GenBank LC586433.1) consisted of 495 base pairs (bp) and was predicted to encode 164 amino acids. Canine p21 protein showed 82% homology with the human p21 protein (GenBank NP_000380.1). Canine p21 contained a putative cyclin-dependent kinase inhibitor domain (Figure 6A).

#### 3.4.4. Confirmation of Cross-Reactivity of Anti-Human p21 Antibodies against Canine p21

To confirm the cross-reactivity of the anti-human p21 antibody with canine p21, western blotting was performed using anti-Halo tag and p21 antibodies. Halo-tagged p21 was detected at the same size as anti-Halo and anti-p21 antibodies; thus, the anti-human p21 antibody was demonstrated to have cross-reactivity against canine p21 (Figure 6B).

#### 3.4.5. p21 Expression Was Induced by DNA Damage in CHMp, but Not CHMm

**Figure 7:**
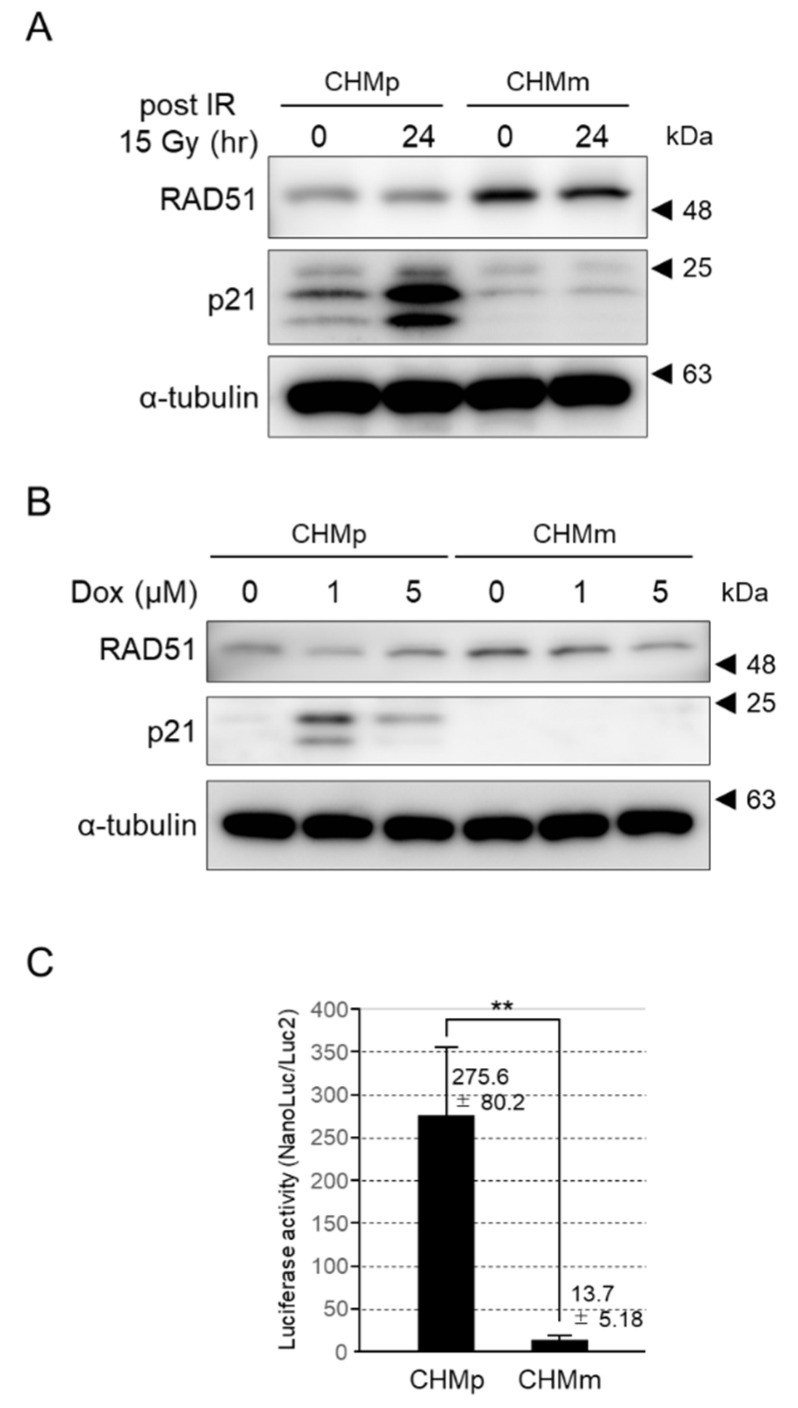
Induction of p21 expression in response to DNA damage, and measurement of transcriptional activity of p21. Expression levels of RAD51 and p21 in CHMp and CHMm after 24 h with 15 Gy irradiation (**A**) and 1 or 5 μM doxorubicin exposure (**B**). α-tubulin was used as a loading control. (**C**) The luciferase activities reflect the p21 transcriptional ability of endogenous CHMp and CHMm cells. The results are given as the mean ± SD. Asterisks on top of the brackets indicate significant differences calculated using ANOVA with Tukey’s multiple-comparison test (** *p* < 0.01). (The original gel figures can be found in the Supplementary Materials.).

To verify why CHMm cells with high RAD51 expression were vulnerable to DNA damage, we examined p21 expression in cells induced by DNA damage. In both CHMp and CHMm cells irradiated with 15 Gy, RAD51 expression was unchanged before and 24 h after irradiation; however, p21 expression increased only in CHMp (Figure 7A). Similarly, p21 expression was increased in CHMp cells at 24 h after exposure to 1 or 5 µM doxorubicin, but not in CHMm cells (Figure 7B). The increase in p21 expression in CHMp cells was maximal at 1 µM doxorubicin exposure under these experimental conditions.

#### 3.4.6. The p21 Transcriptional Activation of CHMm Cells Was Significantly Reduced

The p53 tetramer response element, along with the human cytomegalovirus minimal promoter sequence, was cloned upstream of the sequence encoding Nano Luc [26], and the resulting plasmid was transfected into CHMp and CHMm cells to measure endogenous p21 transactivation abilities. CHMm cells showed an approximately 20-fold reduction in luciferase activity compared to CHMp cells (Figure 7C).

## 4. Discussion

Mammary tumors are a common disease in dogs, and a variety of treatment options are available [32,33], including radiation therapy. The combination of radiation therapy with PARP inhibitors for cases of BRCA2-mutated canine mammary tumors is also being studied [32]. The DNA damage repair system of tumor tissues is key to the success of radiotherapy aimed at DNA lesions in tumor cells [34]. HR repair is the primary repair system for irradiation-induced DNA DSBs. RAD51 [5], which is closely involved in the HR process, is often overexpressed in chemoresistant radioresistant human tumor tissues [35,36]. Increased expression of HR-related molecules, such as RAD51 and BRCA2, has also been observed in canine mammary tumor tissue [18,19], but their functional roles in tumor cell lines have not been investigated.

Comparison of RAD51 expression in eight cell lines derived from mammary tumors using western blotting in G_0_ and S phases synchronized using double thymidine block revealed that the expression levels varied among cell lines, but no differences were observed between cell cycles. This is because RAD51 does not exhibit increased protein quantity upon DNA damage, but functions by congregating at the site of damage [37,38]. In this study, we selected CHMp and CHMm, which are derived from the same individual and showed marked differences in RAD51 expression using western blotting analysis and compared their radiosensitivity. After irradiation from 0 to 15 Gy, no difference in the survival rate of both cells as indexed by IC_50_ up to 5 Gy irradiation was observed. However, at 10 and 15 Gy irradiation, the survival rate decreased in CHMm cells having higher RAD51 expression. When cells were scratched 72 h after 15 Gy irradiation, the recovery rate at 24 h was significantly higher for CHMp. These results indicate that CHMm with higher RAD51 expression is more radiosensitive than CHMp with lower RAD51 expression.

To investigate the intracellular dynamics of RAD51, which is highly expressed in CHMm cells, we investigated RAD51 nuclear focus formation in cells irradiated with 15 Gy. In CHMm, an internuclear RAD51 was stained deep red at a higher ratio, which was more significant after irradiation. In contrast, the number of CHMp cells with RAD51 internuclear foci increased significantly after irradiation. RAD51 foci after the induction of DNA damage were visualized by RAD51 assembly at DNA damage sites and are an indicator of DNA damage repair by HR [39,40]. Deep-red-stained RAD51 in the internuclear region of CHMm indicates a high expression of RAD51, but this may not be a functional congregation.

When cells are exposed to DNA damage, tumor suppressor genes, such as p53, are expressed, resulting in cell cycle arrest [41]. FACS analysis was performed to confirm the cell cycle stages of CHMp and CHMm cells after irradiation. CHMp synchronized to the G0 phase showed a doubling of the DNA content index at 6 h under non-irradiated conditions, but the doubling cells were reduced in the 15 Gy irradiated group. However, a DNA content index of less than two-fold but more than one-fold was observed even in cells 6 h post-irradiation, suggesting simultaneous DNA replication and cell cycle arrest in CHMp cells. In contrast, in CHMm cells, the DNA content index doubled at 6 h in both the non-irradiated and irradiated groups. This suggests that cell cycle arrest may not have occurred in the CHMm cells. Although CHMp and CHMm cells were derived from the same individual, they showed differences in the DNA content index at the G0 phase after the double thymidine block. Therefore, we performed a microsatellite analysis to confirm the clonality of these cells. Thirteen microsatellite markers were evaluated, and differences were observed in four loci between CHMp and CHMm cells. Because microsatellite marker analyses of mammary tumor cell lines derived from the same individual in previous studies in dogs showed differences of two–five loci among cell lines, the present results are not considered abnormal for the genomic status of cell lines derived from the same individual [26].

Because irradiation suppressed cell cycle progression in CHMp, but not in CHMm, we examined the intracellular dynamics of p21, a p53 downstream molecule, and a major cell cycle regulator during DNA damage. Since the canine p21 homolog had not been cloned, it was newly cloned in the present study and the cross-reactivity of the anti-human p21 antibody against canine p21 was confirmed. Structural comparison of canine p21 with human p21 showed high amino acid sequence homology (82%) and conservation of the functional domain of the cyclin-dependent kinase inhibitor. Same-size bands were observed using both anti-Halo and anti-human p21 antibodies against Halo-tag fused canine p21 using western blotting, confirming the cross-reactivity of anti-human p21 antibodies to canine p21. Using this antibody, observation of p21 expression in CHMp and CHMm 24 h after 15 Gy radiation showed that p21 expression was increased in CHMp, but not in CHMm. Furthermore, 1 or 5 μM doxorubicin treatment, which induces DNA damage through reactive oxygen species generation [42], increased p21 expression only in CHMp. This result is consistent with the FACS results showing inhibition of cell cycle progression after DNA damage in CHMp, but not in CHMm. However, the expression level of RAD51 was not changed by radiation, and a slight decrease in expression was observed after 1 μM doxorubicin stimulation. The expression of p21 after DNA damage is triggered by p53 tetramer formation [43]. A nano luciferase reporter assay using the p53 tetramerization response element [26], in which transcriptional activation occurs by tetrameric p53, showed that luciferase activity was approximately 20-fold higher in CHMp expressing p21 than in CHMm not expressing p21. This result suggests that p53 tetramer formation was impaired in CHMm. Since a previous study analyzed the p53 mRNA sequences of eight canine mammary tumor-derived cell lines and did not find mutations in CHMm [26], there may be other reasons for the suppression of p21 expression.

No studies have investigated the radiosensitivity and DNA damage repair mechanisms of canine mammary tumor cell lines by focusing on RAD51 expression profile. Among CHMp and CHMm derived from the same individuals with large differences in RAD51 expression, CHMm with high RAD51 expression was more radiosensitive at high doses of irradiation. It is possible that in CHMm with high RAD51 expression, the rate of RAD51 foci formation during irradiation is low compared to that in CHMp, resulting in inefficient HR. There was also no cell cycle arrest after irradiation in CHMm cells, because p21, a cell cycle regulator, was not expressed. The present study was based on analysis of only one pair of cell lines derived from the same individual. To determine the universal function of overexpressed RAD51 in dogs, more cell types are needed for subsequent investigations. In the future, studies on molecules surrounding RAD51, such as p53, which is involved in p21 expression, and BRCA2, which is essential for the functional expression of RAD51, should be expanded to establish a foundation for elucidating the mechanism of mammary tumorigenesis and developing novel therapeutic strategies in dogs.

## Data Availability

The data presented in this study are available upon request from the corresponding author.

## Supplementary Materials

The following supporting information can be downloaded at: https://www.mdpi.com/article/10.3390/vetsci9120703/s1.

## References

[1] Shinohara A., Ogawa H., Matsuda Y., Ushio N., Ikeo K., Ogawa T. (1993). Cloning of human, mouse and fission yeast recombination genes homologous to RAD51 and recA. Nat. Genet..

[2] Bhat K.P., Cortez D. (2018). RPA and RAD51: Fork reversal, fork protection, and genome stability. Nat. Struct. Mol. Biol..

[3] Sun Y., McCorvie T.J., Yates L.A., Zhang X. (2020). Structural basis of homologous recombination. Cell. Mol. Life Sci..

[4] Schipani F., Manerba M., Marotta R., Poppi L., Gennari A., Rinaldi F., Armirotti A., Farabegoli F., Roberti M., Di Stefano G. (2022). The mechanistic understanding of RAD51 defibrillation: A critical step in BRCA2-mediated DNA repair by homologous recombination. Int. J. Mol. Sci..

[5] Richardson C. (2005). RAD51, genomic stability, and tumorigenesis. Cancer Lett..

[6] Chen J.J., Silver D., Cantor S., Livingston D.M., Scully R. (1999). BRCA1, BRCA2, and Rad51 operate in a common DNA damage response pathway. Cancer Res..

[7] Tarsounas M., Davies D., West S.C. (2003). BRCA2-dependent and independent formation of RAD51 nuclear foci. Oncogene.

[8] Sliwinski T., Krupa R., Majsterek I., Rykala J., Kolacinska A., Morawiec Z., Drzewoski J., Zadrozny M., Blasiak J. (2005). Polymorphisms of the BRCA2 and RAD51 genes in breast cancer. Breast Cancer Res. Treat..

[9] Sharan S.K., Morimatsu M., Albrecht U., Lim D.S., Regel E., Dinh C., Sands A., Eichele G., Hasty P., Bradley A. (1997). Embryonic lethality and radiation hypersensitivity mediated by Rad51 in mice lacking Brca2. Nature.

[10] Qin J., Huang T., Wang J., Xu L., Dang Q., Xu X., Liu H., Liu Z., Shao C., Zhang X. (2022). RAD51 is essential for spermatogenesis and male fertility in mice. Cell Death Discov..

[11] Sleeckx N., de Rooster H., Veldhuis Kroeze E.J., Van Ginneken C., Van Brantegem L. (2011). Canine mammary tumours, an overview. Reprod. Domest. Anim..

[12] Yoshikawa Y., Morimatsu M., Ochiai K., Nagano M., Yamane Y., Tomizawa N., Sasaki N., Hashizume K. (2005). Insertion/deletion polymorphism in the BRCA2 nuclear localization signal. Biomed. Res..

[13] Rivera P., Melin M., Biagi T., Fall T., Haggstrom J., Lindblad-Toh K., von Euler H. (2009). Mammary tumor development in dogs is associated with BRCA1 and BRCA2. Cancer Res..

[14] Hsu W.L., Huang Y.H., Chang T.J., Wong M.L., Chang S.C. (2010). Single nucleotide variation in exon 11 of canine BRCA2 in healthy and cancerous mammary tissue. Vet. J..

[15] Ozmen O., Kul S., Risvanli A., Ozalp G., Sabuncu A., Kul O. (2017). Somatic SNPs of the *BRCA2* gene at the fragments encoding RAD51 binding sites of canine mammary tumors. Vet. Comp. Oncol..

[16] Di Giacomo D., Di Domenico M., Defourny S.V.P., Malatesta D., Di Teodoro G., Martino M., Viola A., D’Alterio N., Camma C., Modesto P. (2022). Validation of AmpliSeq NGS panel for BRCA1 and BRCA2 variant detection in canine formalin-fixed paraffin-embedded mammary tumors. Life.

[17] Yoshikawa Y., Ochiai K., Morimatsu M., Suzuki Y., Wada S., Taoda T., Iwai S., Chikazawa S., Orino K., Watanabe K. (2012). Effects of the missense mutations in canine BRCA2 on BRC repeat 3 functions and comparative analyses between canine and human BRC repeat 3. PLoS ONE.

[18] Klopfleisch R., Gruber A.D. (2009). Increased expression of *BRCA2* and RAD51 in lymph node metastases of canine mammary adenocarcinomas. Vet. Pathol..

[19] Klopfleisch R., Schutze M., Gruber A.D. (2010). RAD51 protein expression is increased in canine mammary carcinomas. Vet. Pathol..

[20] Hu J., Zhang Z., Zhao L., Li L., Zuo W., Han L. (2019). High expression of RAD51 promotes DNA damage repair and survival in KRAS-mutant lung cancer cells. BMB Rep..

[21] Song J., Cui D., Wang J., Qin J., Wang S., Wang Z., Zhai X., Ma H., Ma D., Liu Y. (2021). Overexpression of HMGA1 confers radioresistance by transactivating RAD51 in cholangiocarcinoma. Cell Death Discov..

[22] Wiegmans A.P., Al-Ejeh F., Chee N., Yap P.Y., Gorski J.J., Da Silva L., Bolderson E., Chenevix-Trench G., Anderson R., Simpson P.T. (2014). Rad51 supports triple negative breast cancer metastasis. Oncotarget.

[23] Uyama R., Nakagawa T., Hong S.H., Mochizuki M., Nishimura R., Sasaki N. (2006). Establishment of four pairs of canine mammary tumour cell lines derived from primary and metastatic origin and their E-cadherin expression. Vet. Comp. Oncol..

[24] Michishita M., Akiyoshi R., Yoshimura H., Katsumoto T., Ichikawa H., Ohkusu-Tsukada K., Nakagawa T., Sasaki N., Takahashi K. (2011). Characterization of spheres derived from canine mammary gland adenocarcinoma cell lines. Res. Vet. Sci..

[25] Yoshitake R., Saeki K., Watanabe M., Nakaoka N., Ong S.M., Hanafusa M., Choisunirachon N., Fujita N., Nishimura R., Nakagawa T. (2017). Molecular investigation of the direct anti-tumour effects of nonsteroidal anti-inflammatory drugs in a panel of canine cancer cell lines. Vet. J..

[26] Ochiai K., Azakami D., Morimatsu M., Hirama H., Kawakami S., Nakagawa T., Michishita M., Egusa A.S., Sasaki T., Watanabe M. (2018). Endogenous Leu332Gln mutation in p53 disrupts the tetramerization ability in a canine mammary gland tumor cell line. Oncol. Rep..

[27] Wada S., Van Khoa T., Kobayashi Y., Funayama T., Ogihara K., Ueno S., Ito N. (2005). Prediction of cellular radiosensitivity from DNA damage induced by gamma-rays and carbon ion irradiation in canine tumor cells. J. Vet. Med. Sci..

[28] Tanabe A., Deguchi T., Sato T., Nemoto Y., Maruo T., Madarame H., Shida T., Naya Y., Ogihara K., Sahara H. (2016). Radioresistance of cancer stem-like cell derived from canine tumours. Vet. Comp. Oncol..

[29] Maeda M., Ochiai K., Michishita M., Morimatsu M., Sakai H., Kinoshita N., Sakaue M., Onozawa E., Azakami D., Yamamoto M. (2022). In vitro anticancer effects of alpelisib against PIK3CAmutated canine hemangiosarcoma cell lines. Oncol. Rep..

[30] Blum M., Chang H.Y., Chuguransky S., Grego T., Kandasaamy S., Mitchell A., Nuka G., Paysan-Lafosse T., Qureshi M., Raj S. (2021). The InterPro protein families and domains database: 20 years on. Nucleic Acids Res..

[31] Imagawa T., Terai T., Yamada Y., Kamada R., Sakaguchi K. (2009). Evaluation of transcriptional activity of p53 in individual living mammalian cells. Anal. Biochem..

[32] Thumser-Henner P., Nytko K.J., Rohrer Bley C. (2020). Mutations of BRCA2 in canine mammary tumors and their targeting potential in clinical therapy. BMC Vet. Res..

[33] Valdivia G., Alonso-Diez A., Perez-Alenza D., Pena L. (2021). From conventional to precision therapy in canine mammary cancer: A comprehensive review. Front. Vet. Sci..

[34] Shah C., Bauer-Nilsen K., McNulty R.H., Vicini F. (2020). Novel radiation therapy approaches for breast cancer treatment. Semin. Oncol..

[35] Vispe S., Cazaux C., Lesca C., Defais M. (1998). Overexpression of Rad51 protein stimulates homologous recombination and increases resistance of mammalian cells to ionizing radiation. Nucleic Acids Res..

[36] Xu Y., Chen K., Cai Y., Cheng C., Zhang Z., Xu G. (2019). Overexpression of Rad51 predicts poor prognosis and silencing of Rad51 increases chemo-sensitivity to doxorubicin in neuroblastoma. Am. J. Transl. Res..

[37] Yuan S.S., Chang H.L., Lee E.Y. (2003). Ionizing radiation-induced Rad51 nuclear focus formation is cell cycle-regulated and defective in both ATM(−/−) and c-Abl(−/−) cells. Mutat. Res..

[38] Demeyer A., Benhelli-Mokrani H., Chenais B., Weigel P., Fleury F. (2021). Inhibiting homologous recombination by targeting RAD51 protein. Biochim. Biophys. Acta Rev. Cancer.

[39] Daboussi F., Dumay A., Delacote F., Lopez B.S. (2002). DNA double-strand break repair signalling: The case of RAD51 post-translational regulation. Cell Signal..

[40] Laurini E., Marson D., Fermeglia A., Aulic S., Fermeglia M., Pricl S. (2020). Role of Rad51 and DNA repair in cancer: A molecular perspective. Pharmacol. Ther..

[41] Engeland K. (2022). Cell cycle regulation: p53-p21-RB signaling. Cell Death Differ..

[42] Thorn C.F., Oshiro C., Marsh S., Hernandez-Boussard T., McLeod H., Klein T.E., Altman R.B. (2011). Doxorubicin pathways: Pharmacodynamics and adverse effects. Pharmacogenet. Genom..

[43] Gencel-Augusto J., Lozano G. (2020). p53 tetramerization: At the center of the dominant-negative effect of mutant p53. Genes Dev..

