# Supplementary figures and images for: Canine Mammary Tumor Cell Lines Derived from Metastatic Foci Show Increased RAD51 Expression but Diminished Radioresistance via p21 Inhibition
Source: Vet Sci. 2022 Dec 17;9(12):703. doi: 10.3390/vetsci9120703 (PMC9784702; doi:10.3390/vetsci9120703)

Figure 1

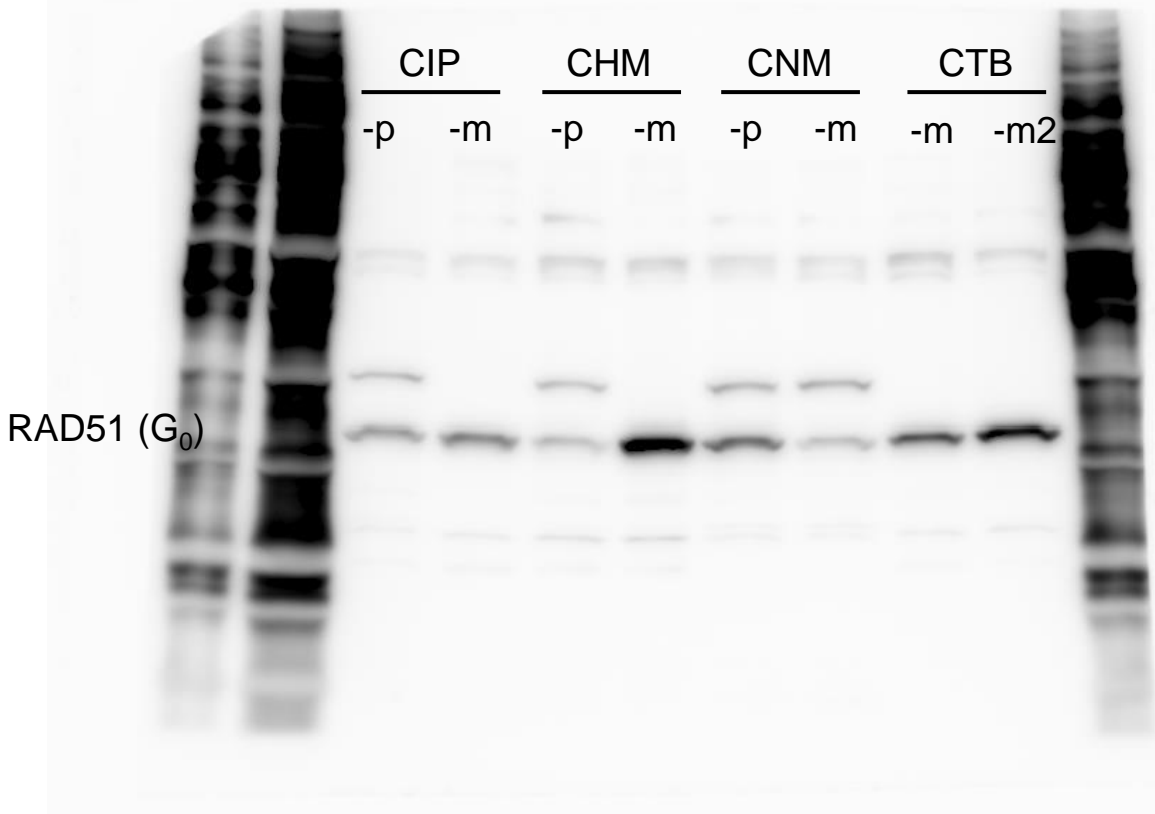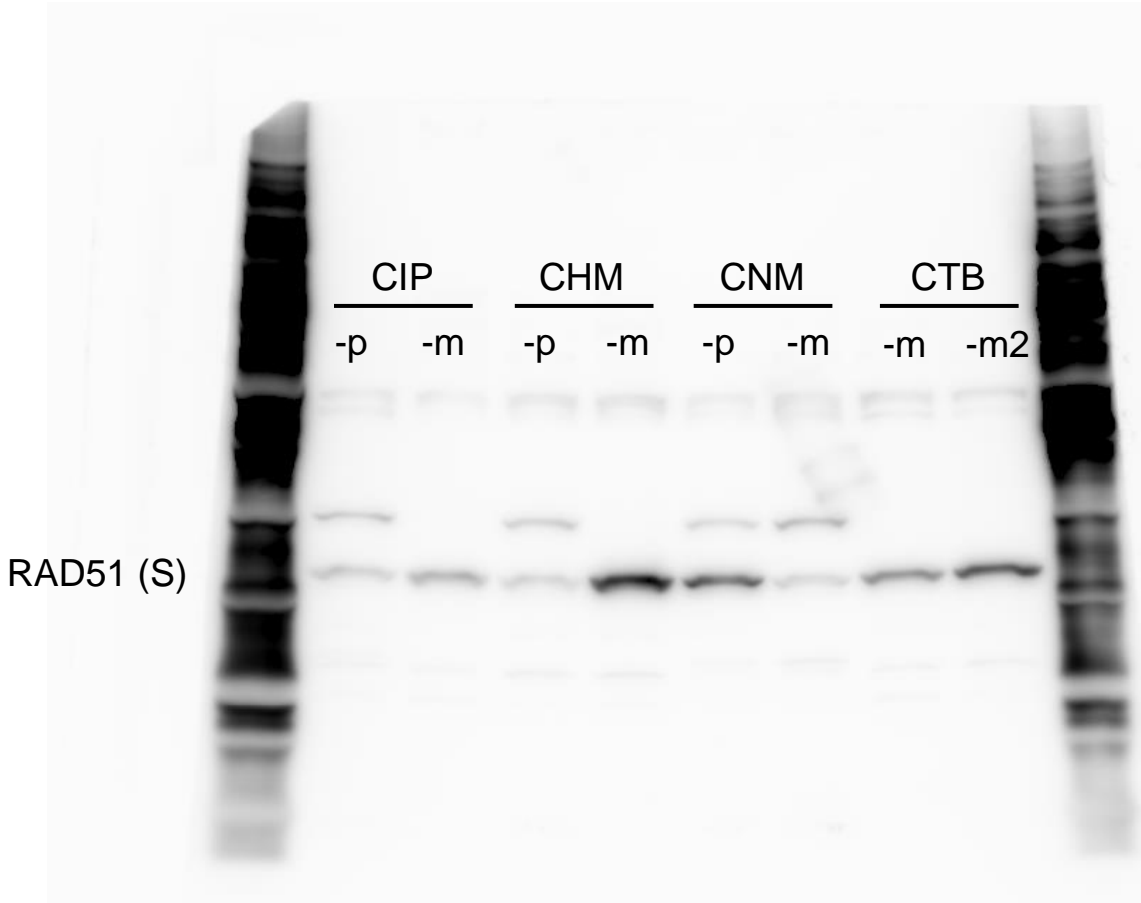

Figure 1

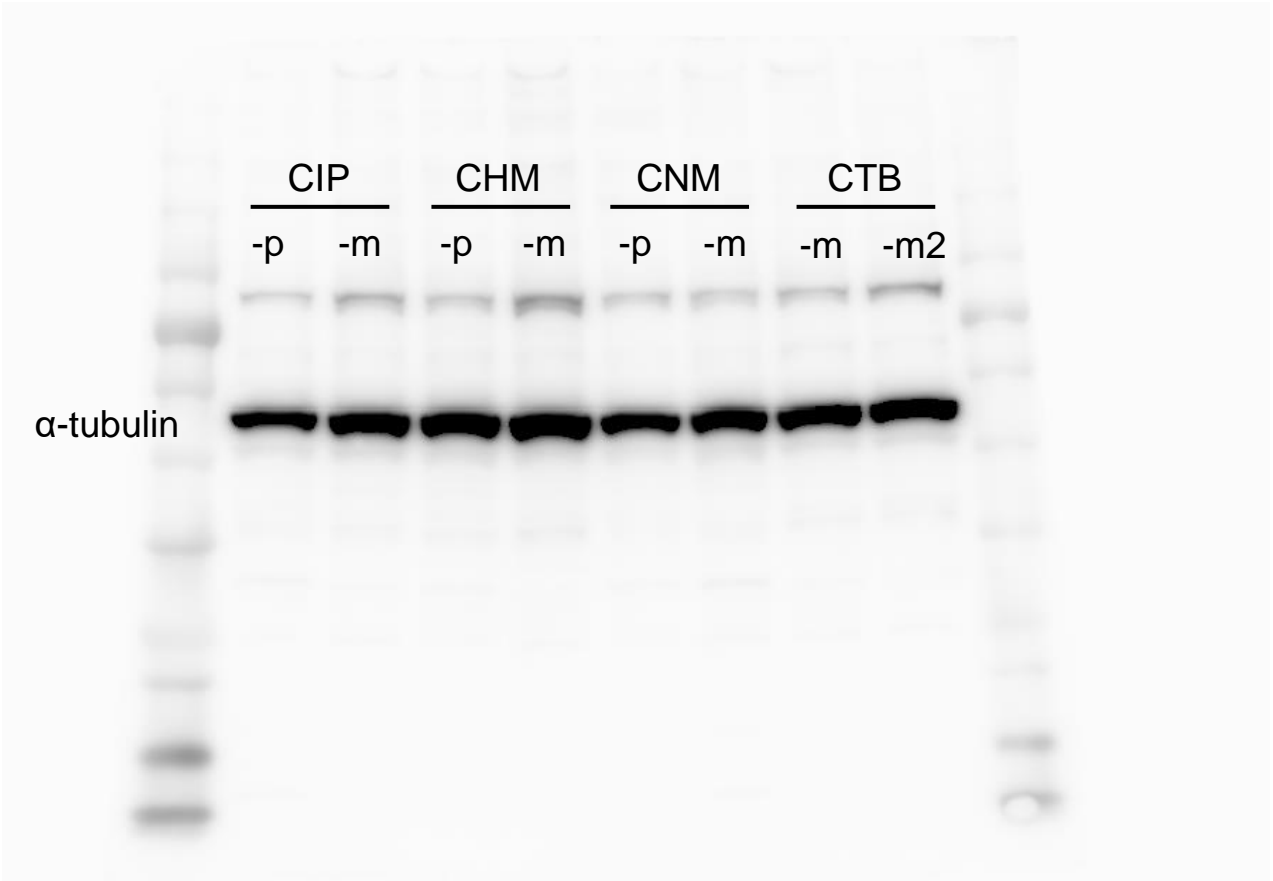

Figure 6

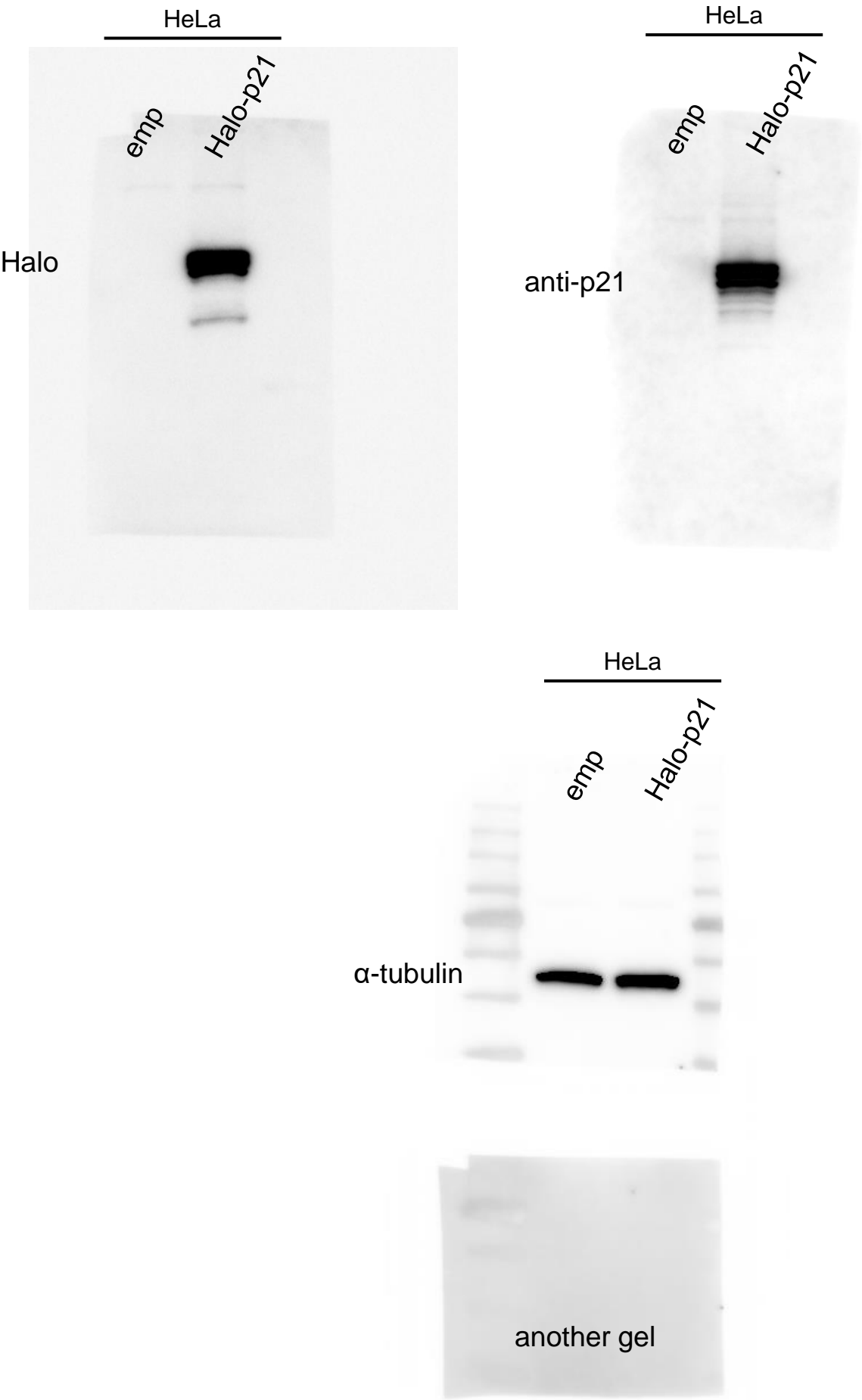

Figure 7A

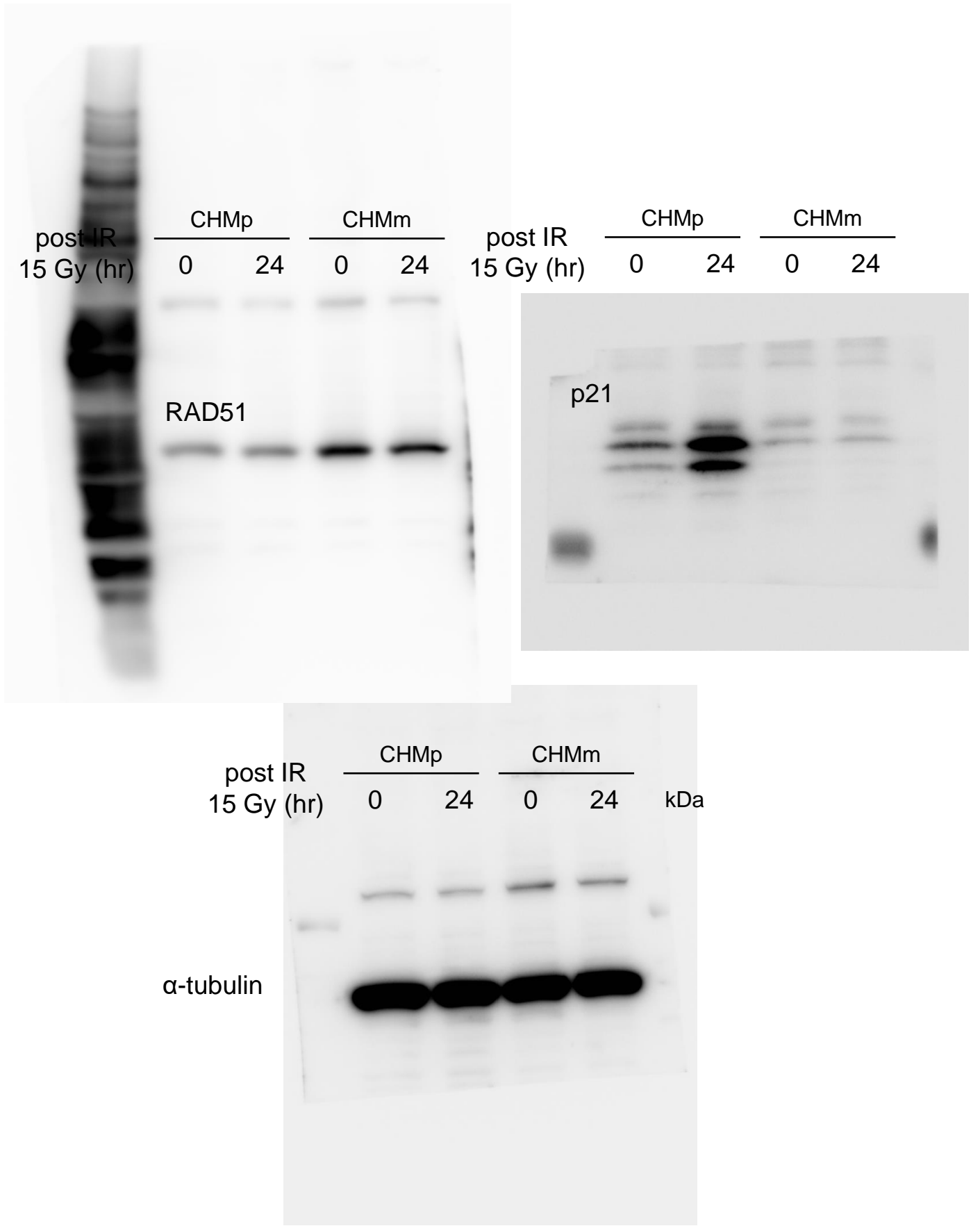

Figure 7B

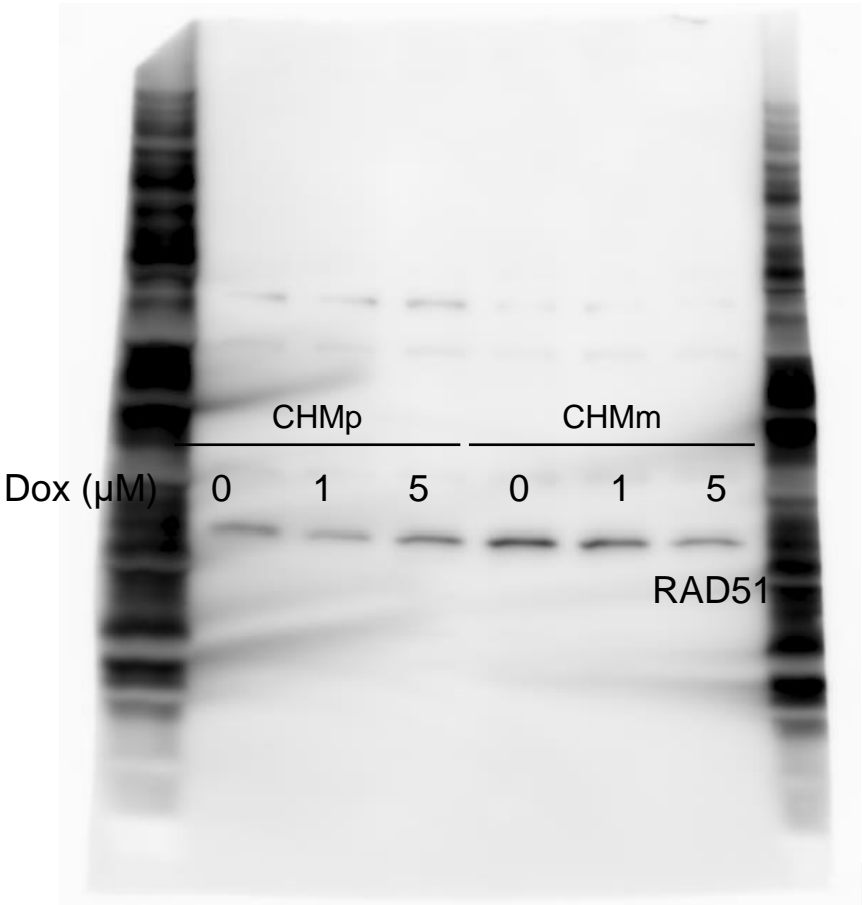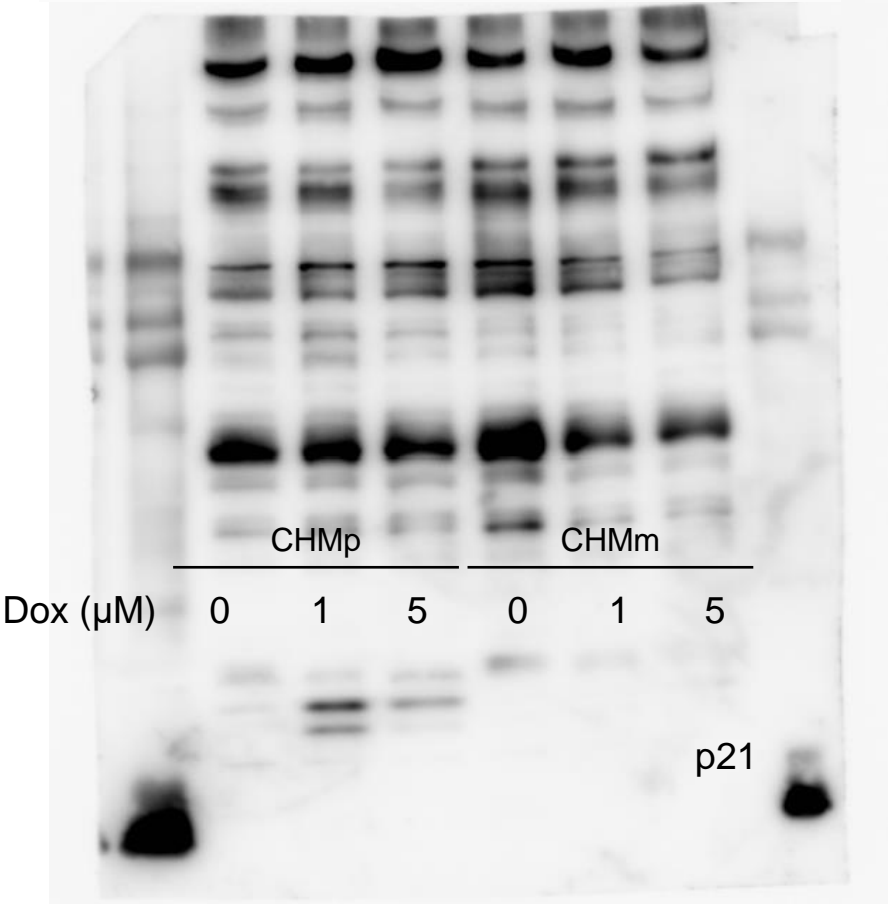

Figure 7B

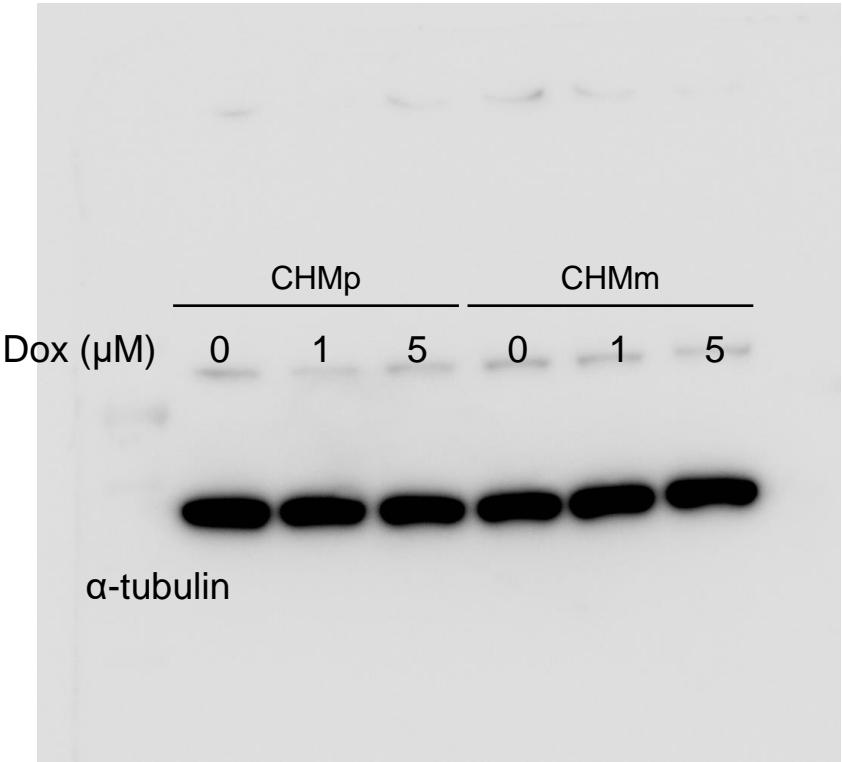

Supplement: Supplementary file 1 [file vetsci-09-00703-s001.zip › vetsci-2079163-supplementary.pdf]
